# Social prescribing by community nurses in southeastern Australia: a qualitative study

**DOI:** 10.1177/17449871261446841

**Published:** 2026-06-05

**Authors:** Jonathan Methley, M Mofizul Islam

**Affiliations:** Community Nurse, Peninsula Health, Melbourne, VIC, Australia; School of Nursing and Midwifery, La Trobe University, Melbourne, VIC, Australia; Department of Public Health, School of Psychology and Public Health, La Trobe University, Melbourne, VIC, Australia; Associate Professor, Department of Public Health, School of Psychology and Public Health, La Trobe University, Melbourne, VIC, Australia

**Keywords:** Australia, community nurse, social aspects of health, social prescribing, social support

## Abstract

**Background:**

Social prescribing involves referring patients to community-based facilities that provide social support. Community nurses can play a crucial role in social prescribing; however, there is limited knowledge about their experiences in this area.

**Aims:**

This study explored the experiences of community nurses in Australia regarding social prescribing.

**Methods:**

Twenty community nurses were recruited and interviewed using snowball and purposive sampling. The nurses held various roles, including crisis management, case manager, team leader, general practice nurse, counsellor and mental health nurse. Thematic analysis was used.

**Results:**

The common social prescribing activities nurses referred to were men’s sheds, coffee groups, walking groups and art therapy. Four themes were identified: (1) a broader view of patient care, (2) barriers to considering social prescribing referrals, (3) barriers to implementing social prescribing referrals and (4) their recommendations. Community nurses prefer social prescribing activities that are affordable and achievable, help clients deal with isolation and keep clients physically active. However, not all nurses were aware of the term ‘social prescribing’.

**Conclusions:**

Community nurses do recognise the social aspects of health. However, they encounter considerable challenges that hinder the implementation of social prescribing. Addressing these challenges is essential for integrating social prescribing into community nursing care.

## Introduction

In most settings, a significant number of primary care patients seek assistance from healthcare providers for issues that are primarily non-medical in nature (Parkinson and Buttrick, 2015; Zimmermann et al., 2018). Providing support for individuals whose health problems are aggravated by non-medical concerns presents a challenge for the healthcare system. Social prescribing activities offer a means for healthcare professionals to refer patients to non-medical/non-clinical services with the aim of improving their health and well-being while at the same time lessening the burden on the primary healthcare system (Woodall et al., 2018). The operation of social prescribing typically involves referrals from healthcare providers, such as general practitioners and nurses, to community-based facilities that provide social activities. By empowering patients and linking them to appropriate community resources, social prescribing aims to promote health and well-being, which may have the potential to reduce the burden on healthcare systems (Drinkwater et al., 2019). A growing number of social prescribing schemes involve link workers or navigators who bridge the gaps between the healthcare professionals and social service facilities (Sharman et al., 2022). Referrals can also be made by medical and non-medical professionals, or patients may self-refer (Islam, 2020).

Social prescribing encompasses a broad range of non-clinical activities that can enhance overall health and well-being by addressing some patient social, emotional and practical needs. At its core, social prescribing connects individuals to local community activities and support groups, which can include social clubs, exercise and fitness classes, art and creative groups, gardening projects and hobby groups. These activities may play a crucial role in fostering social connections, reducing feelings of isolation and providing opportunities for meaningful engagement that contribute to an individual’s mental and physical health (Islam, 2020). Practical support services are also being delivered that include everyday needs such as housing support, financial advice and access to welfare benefits (Husk et al., 2020; Payne et al., 2020; Pescheny et al., 2020).

### Theoretical aspects of social prescribing

Theoretical aspects of social prescribing are primarily grounded in three dominant theories: the theory of salutogenesis, the self-determination theory and the social cure theory (Evers et al., 2024). Salutogenesis, developed by Aaron Antonovsky, focuses on factors that support human health and well-being rather than on the origins of disease (Antonovsky, 1987). This framework introduces two key concepts: sense of coherence and generalised resistance resources. Social prescribing interventions may enhance the sense of coherence, thereby promoting overall health and resilience in individuals. Self-determination theory is a psychological theory of motivation, which explains how the satisfaction of certain psychological needs creates conditions that lead to self-motivation (Ryan and Deci, 2000). Self-determination theory focuses on the process by which individuals gain motivation to initiate and sustain new behaviours. The social cure theory emphasises the importance of social group memberships in promoting health and well-being (Jetten et al., 2012). It posits that belonging to supportive social groups, such as those formed through family, friends and community activities, can significantly improve mental health, reduce stress and enhance overall life satisfaction. The theory highlights key mechanisms like increased social support, improved self-esteem and a stronger sense of identity as contributors to better health outcomes. Social prescribing aligns closely with these theories by facilitating access to social groups and activities, thereby helping individuals build meaningful relationships and a sense of community.

### Social prescribing in Australia

Social prescribing in Australia is currently in its early stages, with limited research and implementation within healthcare settings. Despite this, holistic models are beginning to emerge in primary care, aged care and community services, although there is minimal national oversight (Aggar et al., 2021). Small-scale innovations and trials are underway across the country, indicating a growing interest in this approach (Sharman et al., 2022). Many health practitioners have started to incorporate social prescribing into their daily practices, yet it remains largely unsupported by formal recognition or funding mechanisms (Sandhu et al., 2022). Currently, Australia has no funding model or national framework for social prescribing (Zurynski et al., 2020). However, the Australian government’s National Preventive Health Strategy 2021 to 2030 advocates for the integration of social prescribing to prevent public health concerns (Australian Government, 2021), and Australia’s 10 Year Primary Health Plan 2022–2032 promotes Primary Health Networks to trial models of social prescribing nationally.

As the formal approach to social prescribing is relatively new in Australia, there is limited evidence of this scheme. Lack of high-quality evidence, like in many other settings, is a barrier to the expansion of social prescribing. Early studies have shown some positive results regarding the usefulness of social prescribing. For instance, a pilot study by Aggar et al. (2021) evaluated a social prescribing programme aimed at improving the quality of life for participants with mental illness, yielding significant improvements in various outcome measures. Thomas et al. (2020) assessed the impact of Forest Therapy interventions on the quality of life and biopsychosocial well-being of community-living adults with mental health. They found social prescribing could be referred by any healthcare worker and that signposting was not generally effective. Dingle et al. (2022) represented the first controlled evaluation conducted in Australia and found that an absence of high-quality evidence and rigorous theoretical framework were the primary barriers to social prescribing being more widely implemented in Australia. Sharman et al. (2022) examined link workers’ roles and skills required for social prescribing. A recent study found multiple opportunities for social prescribing across the broad range of Australian funding models, and both person- and system-level domains are critical for social prescribing evaluation (Baker et al., 2024). In the existing evidence base, there is little or no information about the perspectives of various healthcare provider groups.

### Community nurses

Community nurses are well-positioned to play a significant role in social prescribing due to their holistic approach to patient care (Kenkre and Howarth, 2018). Their training encompasses not only physical health but also psychological, social and environmental factors that influence overall well-being, aligning closely with the principles of social prescribing. Community nurses often develop long-standing relationships with patients, fostering trust and enabling a deeper understanding of individual needs and preferences (Howarth and Burns, 2019). The advocacy role that nurses play (Choi, 2015) is particularly relevant to social prescribing, as it empowers patients and addresses their specific needs. Additionally, community nurses typically possess extensive knowledge of local resources and services, making them valuable assets in connecting patients with appropriate non-clinical interventions (Chilton and Bain, 2017). Their experience in multidisciplinary teamwork and interdisciplinary collaboration is crucial for the coordination required in social prescribing.

However, there is limited knowledge about the specific practices of social prescribing among community nurses in the Australian context. This gap highlights the need for studies to explore their experiences, and barriers in integrating social prescribing into their practice. Understanding how community nurses engage with social prescribing, including practical challenges and successes, could inform the development of effective implementation strategies. Such research is essential for optimising comprehensive healthcare delivery and enhancing patient outcomes in the Australian healthcare system. Accordingly, this study explored the research question: *How do community nurses in Australia experience and implement social prescribing in their practice*?

## Materials and methods

### Sample and data collection

This qualitative study employed semi-structured and in-depth interviews with 20 current community nurses to explore their experiences of social prescribing. This sample size was considered sufficient to achieve data saturation, whereby no new themes or insights were identified in the later interviews. The recruitment of 20 participants was informed by a systematic review suggesting that saturation in qualitative studies involving relatively homogeneous professional groups is often achieved with 9–17 participants (Hennink and Kaiser, 2022). Participants were recruited through convenience sampling via a flyer displayed at the front desk of the general practices and through word-of-mouth. Interviews were conducted either face-to-face in the workplace or over the phone. No one else was present during the interviews apart from the participants and the interviewer. The study focused exclusively on nurses currently working in community settings, excluding those in hospital wards or no longer in community roles. This criterion ensured a concentrated examination of community nurses’ experiences with social prescribing. Participants were informed about the interviewer’s role, professional background and the purpose of the study.

An interview guide with open-ended questions was employed to encourage in-depth, rich and subjective responses. It covered topics such as participants’ backgrounds, their awareness of social prescribing, whether and how they refer clients to social prescribing activities, the barriers they encounter and their recommendations for improving the practice in Australia. This approach allowed for a comprehensive understanding of participants’ experiences while providing flexibility to explore specific areas of interest. The interview guide was pilot tested prior to data collection. The researchers had received formal training in qualitative research methods and interview techniques, under the supervision of experienced qualitative researchers. The senior researcher is experienced in conducting qualitative studies.

The first author, a community nurse working in Melbourne, Victoria, conducted the interviews. The inclusion criteria included registered nurses currently employed in community settings, residing in Victoria and with English as their primary language. To avoid potential conflicts of interest, the interviewer did not recruit participants from his close social network. Data collection involved the use of two audio recording devices for each interview to enhance reliability. Each interview lasted approximately 30 to 45 minutes, and participants were offered a $40 Coles/Myer gift card as a token of appreciation for their time. The audio recordings were transcribed verbatim and were not returned to participants for checking.

### Data analysed

Data was analysed using the NVivo software (Version 1.7.2). NVivo facilitated the organisation, coding and analysis of complex textual data from interviews of the participants. Its capabilities, including text coding, theme identification and generation of visual representations, were essential for understanding nuanced patterns and trends within the data. The study followed Braun and Clarke’s six-phase thematic analysis framework, which includes (1) familiarisation with data, (2) generating initial codes, (3) searching for themes, (4) reviewing themes, (5) defining and naming themes and (6) writing the report (Braun and Clarke, 2006). The process involved reading audiotaped interview transcripts multiple times before coding. Two coders independently coded each of the 20 transcripts, focusing on the research questions and adjusting codes as necessary. Similar codes were grouped to develop themes, which were then evaluated separately by two researchers to ensure validity. Any conflicts were resolved through face-to-face dialogue. The coding process combined both inductive approaches, designed to capture the details of nurses’ experiences with social prescribing while aligning with research objectives. The analysis followed several steps: initial reading and familiarisation, development of a preliminary codebook, first cycle coding using descriptive and process coding methods, and second cycle coding involving pattern and focused coding. This iterative process involved constant comparison within and across transcripts. Regular discussions with the supervisor were held to review the coding process and emerging themes, enhancing the reliability of the analysis. An example of the coding process and theme development is provided in T﻿able 1. Final themes are outlined in F﻿igure 1.

Trustworthiness was ensured in accordance with Lincoln and Guba’s (1985) criteria. Credibility was supported through regular team discussions and iterative data analysis. Dependability and confirmability were enhanced by maintaining an audit trail documenting data collection, coding decisions and theme development. Reflexivity was maintained throughout, with researchers critically reflecting on their professional backgrounds and potential assumptions. Transferability was facilitated through detailed descriptions of the study context and participants.

### Ethical considerations

This study received ethics approval from the La Trobe University Human Research Ethics Committee (approval number: HEC24149; date: 07/06/2024). In this study, informed consent was obtained verbally from participants at the beginning of each interview. This consent process involved clearly explaining the study’s purpose, procedures, nature of participation and participants’ rights, ensuring that they were fully aware of their involvement and could withdraw at any time without consequence. Confidentiality was rigorously maintained throughout the study. To protect participants’ identities, no names were spoken or recorded during the interviews. Additionally, the handling of data adhered to strict confidentiality protocols, with audio files securely stored and access limited to authorised personnel only with no personal identifiers on the file names. Notably, the study avoided recruiting well-known colleagues to prevent any potential biases or conflicts of interest. Efforts were made to ensure that participants were not coerced into participating, emphasising voluntary involvement and providing a supportive environment for participants to voice any concerns or withdraw if they chose to. This study followed the Consolidated Criteria for Reporting Qualitative Research (COREQ; Tong et al., 2007), and the checklist is provided in the Supplemental Material.

## Results

### Participants’ characteristics

The study involved 20 community nurses in various roles. Nine participants were aged 50–60 years, four were 40–50 years, three were 30–40 years, one was 20–30 years and one was 60–70 years. Briefly, four nurses were crisis assessment treatment team clinicians, three serving as case managers, two acting as team leaders at a local centre, two General Practitioner clinic nurses and one each working as a counsellor/talk therapy nurse, private mental health nurse, intake assessment nurse, alcohol and other drugs counsellor, district nurse, alcohol and other drugs outreach nurse, nurse practitioner for older adults, adult prevention and recovery care nurse and care coordinator. Participants commonly referred clients to activities like men’s sheds, coffee groups, walking groups and art therapy, focusing on affordable and achievable options that address isolation and promote physical activity.

**Table 1.** An example that illustrates the analytical process used to develop the themes.

Data extract	Code	Subtheme	Theme
‘They only fix one part of things . . . they can be a band-aid . . . they don’t fix everything . . . people need to feel a part of the world’.	Medication addresses only part of the problem	Social prescribing addresses the whole person, not just symptoms	A broader view of patient care
‘They don’t fix everything . . . people need to feel a sense of purpose in the world’.	Need for purpose beyond medical treatment	Social prescribing addresses the whole person, not just symptoms	A broader view of patient care
‘Medication is part of the solution, not the full solution . . . we need to promote independence and community integration’.	Holistic rather than symptom-focused care	Social prescribing addresses the whole person, not just symptoms	A broader view of patient care
‘Recovery in a group . . . doing something . . . achieving something . . . impacting their confidence and self-esteem’.	Social participation improves confidence and recovery	Social prescribing addresses the whole person, not just symptoms	A broader view of patient care
‘People with mental health problems or trauma end up being socially isolated . . . once they challenge that and give it a go it’s often a good experience’.	Addressing isolation through social engagement	Social prescribing addresses the whole person, not just symptoms	A broader view of patient care
‘You see some people have lost the will to live . . . they’ve got no quality of life’.	Focus on quality of life beyond symptom control	Social prescribing addresses the whole person, not just symptoms	A broader view of patient care
‘You don’t want them sitting in their wheelchair watching TV all day . . . you want them to have social interaction’.	Promoting active social life for well-being	Social prescribing addresses the whole person, not just symptoms	A broader view of patient care
‘If you look at the biological model it just addresses neurotransmitter deficiency . . . social prescribing addresses practical issues people experience in life’.	Biomedical model is limited to biological factors	Social prescribing addresses the whole person, not just symptoms	A broader view of patient care
‘Many people have tried the biomedical model previously and it hasn’t worked . . . so we branch out to social prescribing’.	Alternative approach when biomedical care is insufficient	Social prescribing addresses the whole person, not just symptoms	A broader view of patient care
‘For people it’s about connection . . . isolation and loneliness predict mental health’.	Social connection as a determinant of well-being	Social prescribing addresses the whole person, not just symptoms	A broader view of patient care
‘People present with social crises . . . job loss, financial stress, lack of support . . . referring them to community services helps relieve mental health pressure’.	Addressing social determinants of distress	Social prescribing addresses the whole person, not just symptoms	A broader view of patient care
‘Medication is part of the solution, not the full solution . . . we need to promote independence and community integration’.	Holistic care, independence and community integration	Social prescribing addresses the whole person, not just symptoms	A broader view of patient care

### Themes

**Figure 1.**
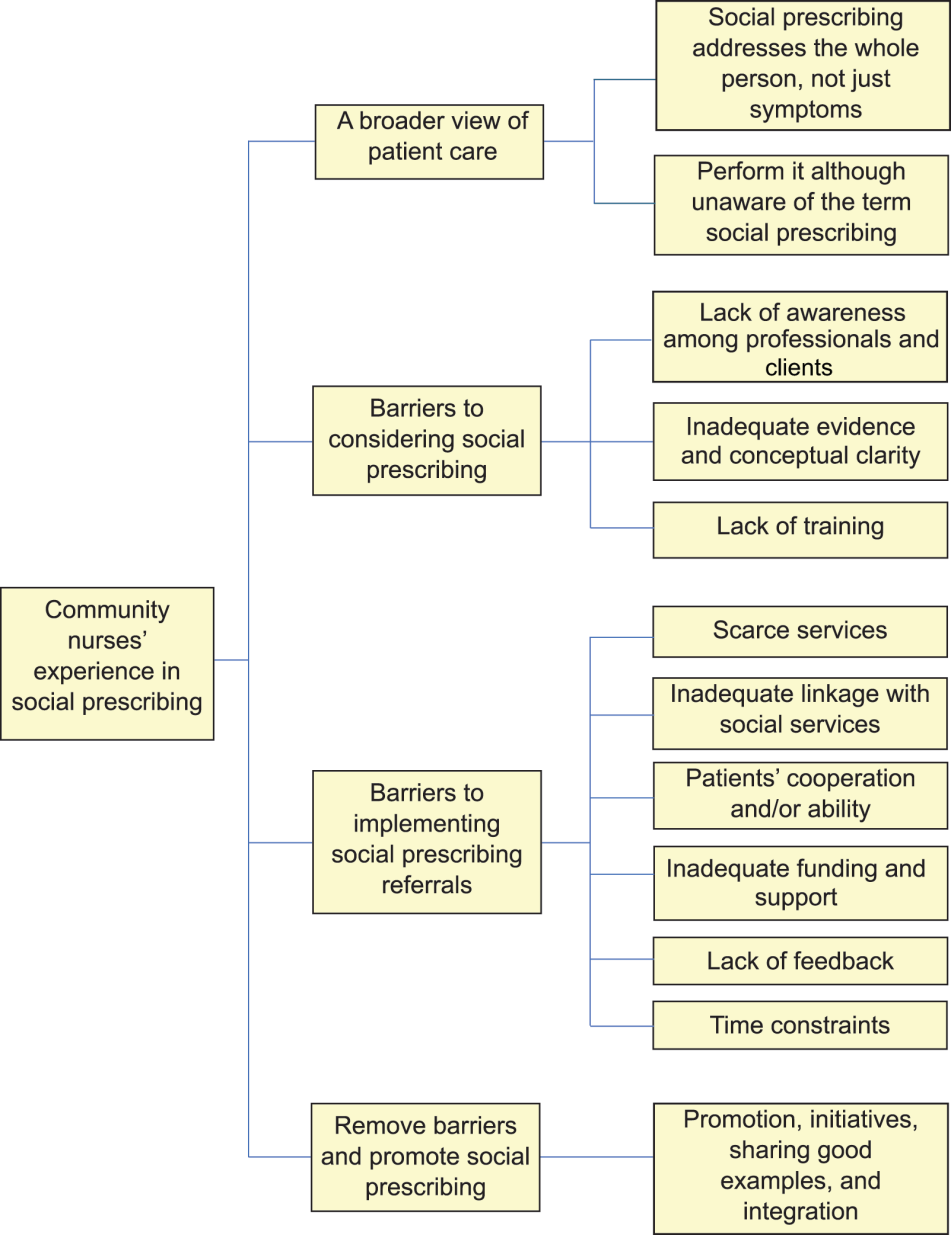
Community nurses’ experience in using social prescribing.

Thematic analysis identified four major themes: a broader view of patient care, barriers to considering social prescribing, barriers to implementing social prescribing referrals and recommendations. These themes along with sub-themes are presented in Figure 1 and described below.

#### A broader view of patient care

##### Social prescribing addresses the whole person, not just symptoms

Nurses identified biomedicine as addressing a limited aspect of health. Although biomedicine plays a crucial role, it should not be the sole focus of healthcare. The psychosocial component is also regarded as important as biomedicine.

. . .we do have to look at people holistically and, you know, health isn’t just clinical. And often, you know, particularly for people living out in the community and those that are on their own, you know, they become socially isolated, they become depressed, which has a knock-on effect of leading to clinical issues, you know, such as depression, decreased mobility. … which then leads to pain and, so on and so forth. So it’s a bit of a downward spiral and they become frail. Yeah, so, you know, it keeps the mind active as well. (Interviewee 13, age 50)

##### Perform social prescribing although unaware of the term

The term social prescribing was not known by all the community nurses, yet almost all of them practice it. The concept of holistic care was frequently mentioned, emphasising the importance of considering health in its entirety rather than relying solely on biomedicine.

I wasn’t aware of that term particularly. If it was called holistic health, that’s how I would have . . . yeah. (Interviewee 12, age 54)

#### Barriers to considering social prescribing referrals

##### Lack of awareness among professionals and clients

A main barrier discussed was the lack of awareness of the concept of ‘social prescribing’ amongst professionals and their clients. This basic knowledge of social prescribing would encourage and empower nurses to incorporate it into their practice and inform and explain to their clients what social prescribing entails.

I think it’s a matter of the term becoming more widespread, everyone understanding what social prescribing is, and I think it also has to be a change that occurs within the system. (Interviewee 17, age 30–40)

##### Inadequate evidence and conceptual clarity

With social prescribing becoming a relatively new phenomenon in Australia, evidence is required to encourage nurses to see the benefits and practice social prescribing. The following quote substantiate this observation:

I think, you know, having access to evidence-based literature that shows that it, it works and that it, it’s, it’s a really powerful tool and something that you can share with a client about, you know, this is the impact this can have on you. Not just go and join the coffee club because you need to get out, which can sort of seem a bit judgmental. (Interviewee 2, age 50–60)

##### Lack of training

The lack of training in both university and healthcare settings was identified as a barrier to the uptake of social prescribing. Nurses acknowledged that up-to-date knowledge is important, which highlights the importance of training or even inclusion of social prescribing in the nursing curriculum.

I’ve taught at university as well, and it’s not something that’s really discussed. Like, talk about, you know, biopsychosocial model and holistic care and things like that. Um, but to be honest, I haven’t, like, myself as a student and then teaching, I haven’t heard it really discussed much as an option (Interviewee 10, age 36)

#### Barriers to implementing social prescribing referrals

##### Scarce social prescribing activities

One of the main perceived barriers was the scarcity of social prescribing activities to which patients could be referred, with the unavailability and long wait times for the few schemes that offer social prescribing forced nurses to have to rely on referring back to clients’ general practitioners. A community nurse stated:

I think availability is a key issue. The thing that puts us off the most is that there are limited services out there. Thus, we have insufficient access to services and lots of long waiting lists, and so I think more people are inclined to send them (patients) back to the general practitioners and hope that the general practitioners can link them into something instead (Interviewee 18, age 47)

##### Inadequate linkage with social services or activities

A lack of effective connection with social prescribing activities was identified as a barrier. Although there are some existing activities, insufficient links with these programmes restrict their ability to refer clients. The location of both the clients and the available resources plays a crucial role in this poor connectivity. As a result, nurses have limited referral options for their patients.

Particularly say if we’re looking on the peninsula, we’re a big area and maybe the services are more located into the central business districts. Whereas in the regional or more rural parts of the peninsula, they don’t necessarily have as many social supports networks that we can link people in with (Interviewee 6, age range 50–60)

##### Patients’ cooperation and/or ability

Nurses reported that not all clients were willing to participate in social prescriptions. Some patients may encounter challenges, such as lack of transportation for attending referrals and social prescribing.

I would say the number one barrier is often the client themselves. If they don’t feel, with your recommendations or ideas, sometimes they just don’t, there may be some, so unwell or in a space where they’re just not ready to do such things, so first of all the client maybe a barrier to themselves, sometimes location as well sometimes people really struggle you know because we’re targeting a group where people are often financially struggling and you know often don’t have transport. (Interviewee 8, age range 40–50)

##### Inadequate funding and support

Funding was emphasised to ensure smooth referrals and availability of social activities for patient referrals. A small number of participants indicated that this shortage of funding could be attributed to the National Disability Insurance Scheme (NDIS) budget. Here are the opinions of two participants:

I think that we need to do more as a whole. I think we did well many years ago and I think it’s just fallen on the wayside due to resources and money in the services. I think that’s where it’s all fallen down. And also the government giving it all to the NDIS. I think that that hasn’t worked. I think that needs to be reviewed and that’s my opinion. (Interviewee 15, age 57)

##### Lack of feedback

Poor communication among key stakeholders about the outcomes of patient referrals discourages some nurses from referring patients to social prescribing programmes. A lack of feedback creates barriers to understanding the effectiveness of these programmes and adjusting referrals as needed. Nurses have emphasised the importance of receiving feedback on patient adherence to the interventions and their effectiveness. The following statement supports this point:

The referrals are a very long and drawn out process. But yeah, and as a referrer, because I do a lot of referrals to my aged care, once I’ve sent that referral off, I don’t get any feedback. So, I don’t know what’s happened next. And if I try and call my aged care and ask them, they’ll tell me I’m not authorised and the client has to call themselves. (Interviewee 18, age 47)

##### Time constraints

Nurses often struggle to dedicate time to engage in social prescribing activities. Some of them thought that the enterprise bargaining agreements should mandate specific work hours for social prescribing duties. This lack of protected time hinders the ability of nurses to fully support patients in accessing community-based resources, ultimately limiting the effectiveness of social prescribing interventions.

So more often than not we run at numbers that are above our equivalent full-time (EFT) and we don’t have time to really spend a lot of one-on-one time. Essentially the best way to do this would be to either allow, I suppose in the enterprise bargaining agreement (EBA), some mandated social prescribing time or mandated psychosocial recovery time where you have protected slots to go out and see clients, similar to supervision perhaps, but also just more, you know, more people at the call face. (Interviewee 17, aged 30-40)

#### Remove barriers and promote social prescribing

##### Promotion, initiatives, sharing good examples and integration

All nurses wanted to remove barriers that hindered them from referring clients to much-needed social activities. Participants recommended promoting social prescribing not only among nurses but also in the community as a whole.

I guess the councils and the community need to be a little bit more involved about what’s out there… and give greater access, I guess, to healthcare professionals and carers and, you know, and the community itself as to what is actually available. Because often you don’t know about something until somebody actually tells you. It’s very disjointed, you know, the information’s all over the place. (Interviewee 13, age 50)

Nurses voiced that successful initiatives must highlight how connecting patients to non-clinical community resources can enhance well-being, reduce healthcare demand and address some downstream elements of social determinants of health – all of which can come in the form of sharing good examples, such as collaborations between healthcare providers.

I guess it always helps when we hear of positive outcomes of participants that have gone through the social prescribing side rather than the medical model and what a change they have seen. Those stories always hit home. Feedback on certain services is really important from other staff members that have been there or had participants saying how positive the experience was. (Interviewee 5, age 40)

## Discussion

This study examined the experiences of southeastern Australian community nurses with social prescribing. Community nurses often refer their patients to community-based social services, although some may not be familiar with the term ‘social prescribing’. Common forms of social prescriptions included men’s sheds, coffee groups, walking groups and art therapy. Nurses reported several barriers to effective social prescribing, including a lack of available social services, time constraints and poor integration between healthcare and community services. These challenges align with existing literature and highlight the need for multisectoral approaches to address them. The desire of nurses to move beyond a purely biomedical model is consistent with modern healthcare trends, but systemic issues must be resolved for successful implementation. Overall, this research offers valuable insights into social prescribing practices in Australia and identifies areas for further investigation and improvement.

A subset of community nurses was unaware of the concept social prescribing and/or not fully informed about the evidence supporting this initiative. Social prescribing is relatively new phenomenon in Australia, which may have contributed to this knowledge gap. Even in the United Kingdom, where social prescribing is well established, many nurses were unaware of these activities (Nieves, 2019). Additionally, the scientific evidence supporting social prescribing is only gradually emerging (Lynch et al., 2025). Literature consistently suggests that healthcare providers often highlight the importance of developing more robust evidence on social prescribing, albeit they acknowledge the challenges of conducting rigorous research in community settings (Aughterson et al., 2020a). There are very few robust, well-designed long-term evaluations of the impacts of social prescribing on patients, healthcare needs and utilisation, and with general practitioners, nurses and other health professionals, and on link workers and community services.

The study identified several other barriers and challenges that the community nurses face when then practice of social prescribing. Most of these are consistent with those reported in the literature, including issues related to funding, shared understanding, patient disengagement, lack of partnerships, absence of link workers or navigators (Aughterson et al., 2020b; Pescheny et al., 2018), limited formal training, time constraints (Aughterson et al., 2020b), low public awareness of social prescribing (Cooper et al., 2024; Sharman et al., 2022) and ongoing conceptual ambiguity (Cooper et al., 2024). Additionally, many of the barriers faced by community nurses, such as limited services, inadequate connections with social services, insufficient funding and support and a lack of training on social prescribing, are beyond the remit of the community nurses. In the United Kingdom, the National Health Service (NHS) plays a crucial role in fostering a supportive environment for social prescribing (Pescheny et al., 2020), through cross-sector working practices, involving practitioners, commissioners, social prescribers, academics and members of the public (Wallace et al., 2024). Likewise, in Akita Prefecture, Japan, a pilot project on social prescribing brought together the government, the Akita Medical Association, an academic department of Akita University and other relevant organisations (Ota et al., 2025). Similar proactive initiatives are required from Government – both federal and state Departments of Health in Australia – in partnership with academic institutions and local third sector organisations to effectively promote and expand social prescribing.

Our study identified feedback on the outcome of users from using social prescribing activities as a significant barrier. This issue is occasionally overlooked in the literature. However, our findings are consistent with a study that also emphasised the importance of feedback from social prescribing facilities (Kenkre and Howarth, 2018). This feedback is crucial because it allows both patients and providers – such as community organisations – to make necessary adjustments to referrals, address emerging challenges and enhance collaboration and partnership (Chatterjee et al., 2018; Pescheny et al., 2018). Most importantly, without this feedback, nurses who refer patients to social prescribing services lack insight into patients’ uptake, adherence and the overall benefits they receive.

The desire among nurses to move beyond a strictly biomedical model aligns with modern healthcare trends emphasising the complete care of their patients. They refer their patients to social services, although many of them were unaware of the concept of social prescribing. Indeed, some health and social care professionals have been practicing social prescribing for centuries, although this concept has gained prominence lately as its advocates have tried to semi-formalise the process (Islam, 2025). This observation is consistent with the literature, which suggests that in many settings, healthcare providers acknowledge the importance of non-clinical services for the overall health and well-being of their patients and try to address them on an ad hoc basis (Islam, 2020). Establishing a formal system for referring patients could enhance the number of referrals and improve the perceived value of these referrals. Only one nurse indicated that a link worker manages their referrals, suggesting that other referrals may be left to the patients themselves or to social service organisations. The presence of a link worker could help improve adherence, increase the number of referrals and facilitate feedback regarding the outcomes of social prescribing (Baker et al., 2024; Baska et al., 2021; NHS, 2022; Nowak and Mulligan, 2021).

### Implications

Given that this study is an initial exploration of community nurses’ involvement in social prescribing in southeast Australia, the findings represent just a small segment of the broader landscape. Many aspects of social prescribing remain unexplored, and further research is warranted. Although all healthcare providers can refer their patients to social prescribing activities, referrals appear to be made predominantly by general practice professionals. Notably, only three participants in this study were currently working in general practice settings. Future research should broaden the scope and include a wider range of practitioners, such as nurse practitioners, practice nurses, care planning nurses and general practitioners. In addition, only one participant reported close involvement with a link worker, highlighting the need for further research to examine differences between practitioners with and without link worker support.

Nursing education and practice should systematically integrate social prescribing by embedding its principles, referral pathways and community resource mapping into curricula and continuing professional development. Skills-based training, particularly in communication, motivational interviewing and person-centred assessment, can equip nurses to identify non-medical needs and make appropriate referrals. Enhanced interprofessional collaboration with community organisations can further improve coordination and impact.

At the healthcare system and policy level, stronger institutional and regulatory support is needed to embed social prescribing within routine care. This includes the development of clear policy frameworks, sustainable funding models and appropriate workforce structures. While many nurses and other healthcare providers already incorporate social prescribing into their daily practice, it remains insufficiently recognised within existing health system structures. Strengthening awareness of local services, clear referral pathways, data systems and outcome monitoring mechanisms should be established to ensure consistency, accountability and evidence-informed practice. Policies should also prioritise intersectoral collaboration between healthcare and community services to strengthen referral networks (World Health Organization, 2022).

### Strengths and limitations

The study presents several strengths and limitations. To our knowledge, this is the first research of its kind to explore the experiences of community nurses regarding social prescribing in any region in Australia. We made an effort to recruit nurses from a variety of community settings, which is another strength of this research. Additionally, the nursing background of the interviewer facilitated effective interaction, rapport-building and communication during the interviews, leading to more in-depth data and richer sharing of experiences related to social prescribing.

However, there are notable limitations. Firstly, we interviewed only two general practice nurses who were directly involved in patient care planning. Although the findings provide valuable insights into participants’ experiences, the transferability of these findings may be limited due to the specific context in which the study was conducted. Readers should, therefore, consider the characteristics of the study population and setting when determining the applicability of the findings to other contexts. Secondly, only half of the potential participants agreed to be interviewed. Although we did achieve data saturation, there remains a possibility that those who did not participate may have had different or even negative experiences. Notably, one of the nurses we approached immediately declined, stating that they did not support social prescribing. Lastly, we acknowledge the potential bias arising from the interviewer’s nursing background, which warrants further considerations, as participants may have felt social pressure to provide responses aligned with what they perceived to be desirable practices regarding social prescribing.

## Conclusion

This study reveals that community nurses in southeastern Australia often adopt holistic care approaches beyond the biomedical model, frequently practicing social prescribing, although some are unfamiliar with the term. However, significant barriers impede its full implementation. To integrate social prescribing effectively into community nursing, a multifaceted implementation approach is needed. This includes enhancing education, raising awareness, engaging stakeholders, expanding service provisions and implementing policy reforms. Further research is crucial to explore targeted interventions and develop strategies to overcome the identified challenges, ultimately improving patient care and outcomes through social prescribing.

**Key points for policy, practice and research**

- This study contributes by exploring community nurses’ experience of social prescribing in their professional practice.
- Although some community nurses are not aware of the term ‘social prescribing’, this study shows that they commonly practice social prescribing.
- Nurses reported several barriers to the uptake and implementation of social prescribing, including conceptual ambiguity and inadequate evidence, lack of social services, time constraints and poor integration between healthcare and community services.
- Policy implications of this review include raising awareness about social prescribing, engaging stakeholders, expanding and integrating service provisions into healthcare.

## Supplemental Material

sj-docx-1-jrn-10.1177_17449871261446841 – Supplemental material for Social prescribing by community nurses in southeastern Australia: a qualitative study Supplemental material, sj-docx-1-jrn-10.1177_17449871261446841 for Social prescribing by community nurses in southeastern Australia: a qualitative study by Jonathan Methley and M Mofizul Islam in Journal of Research in Nursing
